# Crosslinking Multilayer Graphene by Gas Cluster Ion Bombardment

**DOI:** 10.3390/membranes12010027

**Published:** 2021-12-25

**Authors:** Nurlan Almassov, Sean Kirkpatrick, Zhanna Alsar, Nurzhan Serik, Christos Spitas, Konstantinos Kostas, Zinetula Insepov

**Affiliations:** 1 School of Engineering and Digital Sciences, Nazarbayev University, Nur-Sultan 010000, Kazakhstan; nurlan.almassov@nu.edu.kz (N.A.); zhanna.alsar@nu.edu.kz (Z.A.); nurzhan.serik@nu.edu.kz (N.S.); christos.spitas@nu.edu.kz (C.S.); konstantinos.kostas@nu.edu.kz (K.K.); 2 Exogenesis Corp., Billerica, MA 01821, USA; skirkpatrick@exogenesis.us; 3 School of Nuclear Engineering, Purdue University, West Lafayette, IN 47907, USA; 4 Department of Condensed Matter Physics, National Nuclear Research University (MEPhI), 115409 Moscow, Russia

**Keywords:** multilayer graphene, ion beam processing, cross-linking, spectroscopy, TEM

## Abstract

In this paper, we demonstrate a new, highly efficient method of crosslinking multilayer graphene, and create nanopores in it by its irradiation with low-energy argon cluster ions. Irradiation was performed by argon cluster ions with an acceleration energy E ≈ 30 keV, and total fluence of argon cluster ions ranging from 1 × 10^9^ to 1 × 10^14^ ions/cm^2^. The results of the bombardment were observed by the direct examination of traces of argon-cluster penetration in multilayer graphene, using high-resolution transmission electron microscopy. Further image processing revealed an average pore diameter of approximately 3 nm, with the predominant size corresponding to 2 nm. We anticipate that a controlled cross-linking process in multilayer graphene can be achieved by appropriately varying irradiation energy, dose, and type of clusters. We believe that this method is very promising for modulating the properties of multilayer graphene, and opens new possibilities for creating three-dimensional nanomaterials.

## 1. Introduction

There is a current pressing need to develop a highly selective, energy-efficient filter for extremely small substances. Graphene, with its superior properties [1,2,3,4,5,6,7], is a promising candidate for several challenges, including the one above. However, these properties of graphene can be significantly affected by defects introduced into its structure during synthesis or processing [8]. Pores at graphene’s nanoscale can also change its fundamental properties. Nanoporous graphene (NPG) has good potential for the development of an effective membrane for water desalination, natural gas purification, bioprocessing, solvent- and petrochemical-based separations, hemodialysis, and others [9,10,11,12,13,14]. Changes in the properties of graphene, caused by modulating its defects and further obtaining nanopores, can be implemented using several approaches, including particle irradiation [15,16,17], thermal annealing [18,19], chemical reaction [20,21], and deformation treatment [22,23]. Among them, focused beam irradiation has the benefit of obtaining nanopores with a size and density that can be controlled with atomic accuracy at the nanoscale [24,25,26,27,28,29,30,31,32,33]. However, the low efficiency and high cost of this technique make it disadvantageous for wider application [34,35,36].

Another approach involves gas cluster ion beams (GCIB). During contact with the target’s surface, cluster ions interact with many surface atoms simultaneously, and transfer high energy to a very small region, thus creating damaged areas and pores [37,38,39,40,41]. Experimental investigations, including the GCIB irradiation of argon, have been performed so far on suspended and supported monolayer graphene [42]; however, to date, no experimental studies have been carried out on multilayer graphene (MLG).

There are numerous papers with computer simulation results for the bombardment of graphene by gas cluster projectiles, with various kinetic energies and sizes [43,44,45]. Kim et al. [42] considered the cleaning, defect engineering and nanopore milling of suspended graphene with argon clusters. M. Gołuński et al. [44] proposed C60 and Ar projectiles for the controlled perforation of graphene. Others proposed the use of free-standing graphene as a substrate for chemical analysis by secondary ion mass spectrometry (SIMS) [46,47].

In the present study, computational and experimental results of irradiation of graphene and MLG with Ar gas cluster ion beams are presented. The irradiated samples were characterized by Raman spectroscopy and transmission electron microscopy (TEM). We show that irradiation of MLG with argon cluster ions leads to the cross-linking of its layers, and the formation of nanopores.

## 2. Materials and Methods

### 2.1. Multilayer Graphene Synthesis

All initial MLG samples were purchased at Graphene-supermarket. MLG was grown on Cu by means of Chemical Vapor Deposition [48], and then transferred to a copper TEM grid (2000 Mesh) [49]. The CVD process was performed using pure methane as a precursor. A polymer-free transfer method was used to minimize MLG contamination. Typical MLG coverage of TEM grids was 60–90%.

### 2.2. GCIB Process

In the present study, gas cluster ion beams (GCIB) of Ar were used to produce defects on MLG. Irradiation was performed by Ar cluster ions with acceleration energy E ≈ 30 keV (Exogenesis Corp., Billerica, MA, USA) and the total fluence of Ar cluster ions ranged from 1 × 10^9^ to 1 × 10^14^ ions/cm^2^. The technology of gas cluster ion beam irradiation is a unique low-energy method for the surface treatment of ultrathin 2D films. When accelerated clusters interact with the surface of the processed material, the cluster ion does not penetrate deeper than a few atomic layers (≤10 nm). Upon impact on a surface, clusters instantly create extreme transient conditions of temperature and pressure for the surface atoms [48]. Thus, cluster ions are an ideal tool for the large defect fabrication of graphene and other 2D films and, since they do not penetrate deeply into the substrates, defects in the substrate are not created. Therefore, the characterization of defects in the cluster beam irradiated 2D films becomes much easier than for traditional monomer ion beams. Since the GCIB is a “gentle” irradiation technology, the graphene sheets do not begin to fold, wrinkle or curl during processing.

### 2.3. Measurements and Characterization

Raman spectroscopy was used to analyze the evolution of defects in MLG. Samples were studied using the Horiba Lab Ram Evolution system using a helium–neon laser with a wavelength of 532 nm. The beam power was 0.25 mW, and the spot diameter was 1 μm. All spectra were obtained at room temperature.

TEM of samples before and after irradiation was performed at the JEOL JEM 2100 (accelerating voltage 200 kV) and JEOL JEM-1400 Plus facilities (accelerating voltage 120 kV). All samples were taken in light-field TEM.

## 3. Results and Discussion

### 3.1. Molecular Dynamics Simulation

The irradiation of graphene with an argon cluster was studied using the large-scale molecular massively parallel simulator (LAMMPS (https://www.lammps.sandia.gov, accessed on 21 December 2021)) software package. Graphene sheet modeling was materialized through a lattice of four basis atoms in a rectangle crystal cell. Lattice parameters and coordinates of the basis atoms used in LAMMPS are presented in Table 1. Parameters of Tersoff potential are taken from the LAMMPS BNC.tersoff data file, which was then converted to LAMMPS “real” units. Initially, cluster temperature was set to 0 K, simulations were performed in the NVE ensemble, and boundary conditions were periodic in x, y, and z directions. The argon cluster size varied between 10 and 1074 atoms, whereas its incident energy ranged from 2.8 eV/atom to 31 eV/atom in a z-direction, perpendicular to the graphene surface. The graphene sheet size was 1000 × 1000 Å^2^. Tersoff potential was used to describe the carbon–carbon interactions of the graphene sheet, placed in the XY-plane at z = 0, whereas Lennard–Jones and Buckingham potentials were used for Ar–Ar and Ar–carbon interactions, respectively. (See Table 2 and Table 3)

**Figure 1:**
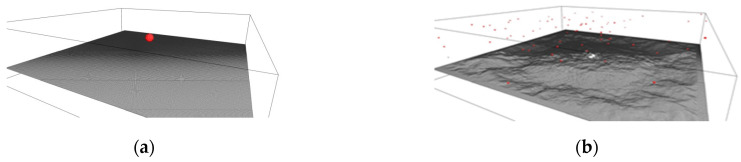
Simulation snapshots of argon cluster bombardment on a graphene sheet at different timesteps: (**a**) 0 fs; (**b**) 100,000 fs.

Figure 1 shows snapshots of our molecular dynamics’ simulations of argon cluster bombardment on a graphene sheet with cluster size of 1074 atoms for graphene areas of 1000 × 1000 Å^2^ at various time instants.

**Figure 2:**
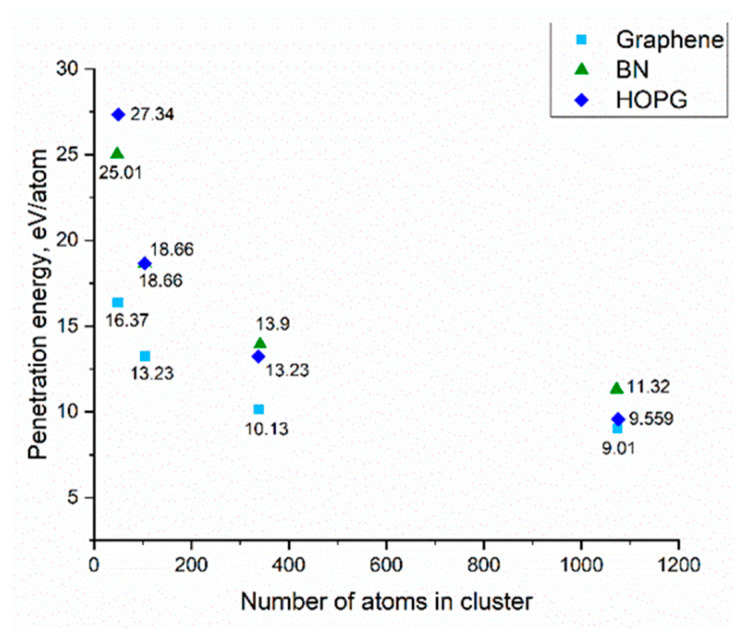
Comparison of the energies of penetration of thin films by an argon cluster ion. The size of the thin film was 1000 × 1000 Å${}^{2}$. The cluster size varied from 42 to 1074 argon atoms per cluster.

Upon irradiation, one can observe defect formation on the graphene sheet, when the incident energy was higher than the threshold value. According to our calculations, the threshold energy of an accelerated argon cluster capable of penetrating and creating a nanopore in graphene was 9.01 eV/atom. Similar calculations for HOPG and boron nitride were 9.559 eV/atom, and 11.32 eV/atom, respectively. The calculation results are shown in Figure 2.

Our simulation results had the same orders of magnitude, and were in good agreement with the values obtained in the work of Zabihi et al. [43]. The pores formed during collisions of an argon cluster with an energy above the threshold with a thin film had diameters ranging from 1–3 nm for a cluster of 102 argon atoms and 10–15 nm for a cluster of 1074 argon atoms. Thus, by adjusting the size and energy of the cluster, it was possible to obtain nanopores with different diameters.

### 3.2. Raman Spectroscopy

**Figure 3:**
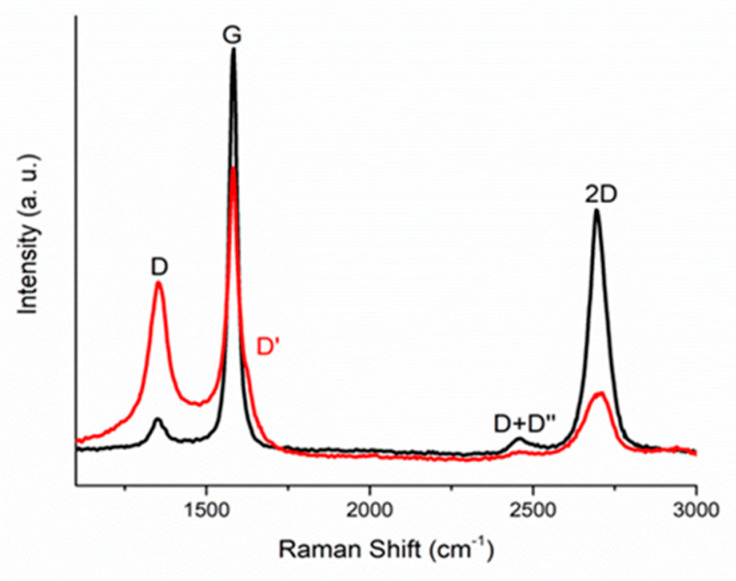
Raman spectra of an unirradiated MLG (black line) and irradiated (red line) MLG by 30 keV (Ar_n_)^+^, where n ≈ 1000, cluster ions at a dose of 10^14^ ion/cm^2^.

Raman spectra of the initial (unirradiated) and resulting (irradiated) MLG samples are shown in Figure 3 with black and red lines, respectively. Four peaks, typical of MLG, can be seen in the depicted spectra. For the initial sample, the D band at about 1350 cm^−1^ appears due to transverse optical phonons at the edges in the Brillouin zone K. This is associated with vibrations of six atomic graphene rings, and requires a defect for its activation. For unirradiated graphene, the D peak was small, which indicated its low defectiveness. The faint band at about 2450 cm^−1^ was attributed to the combination of the D-phonon and the acoustic longitudinal phonon (D′′), and was thus called D + D′′. The G band at about 1590 cm^−1^ was due to first-order Raman scattering by doubly degenerate vibration modes in the plane (plane optical transverse and longitudinal phonons) at the center of the Brillouin zone. The band at about 2680 cm^−1^ appeared due to second-order Raman scattering on plane transverse optical phonons, near the Brillouin zone boundary, and was closely related to the electronic structure of the band.

These four bands were also observed in the Raman spectra of irradiated samples (Figure 3, red curve), but they underwent significant changes. After irradiation with a fluence of 10^14^ cm^−2^, the D’ band appeared at 1620 cm^−1^. This band corresponded to an independent process of intravalley scattering. After irradiation, the intensity of the D band increased significantly, whereas the intensity of the 2D band decreased significantly. The G and D bands overlapped, and the D band broadened due to the coalescence of disordered regions. The ratio of the intensities of the I_D_/I_G_ peaks in irradiated samples was greater than the one in the unirradiated samples, as seen in Table 4. This indicated that the accumulation of defects during irradiation occurred in the MLG itself.

Similar changes were observed in Raman spectra of graphene irradiated with carbon [33] and argon [49] ions, as well as fluorinated graphene [50]. It was shown that the evolution of the Raman spectrum the growth of disorder depends on the type of defect, and this is reflected in the intensities of defect-activated Raman scattering.

**Table 4:** I_D_/I_G_ ratio of graphene.

Sample	I_D_/I_G_	Reference
Unirradiated MLG	0.15	This work
Irradiated MLG	0.6	This work
Ar+bombarded graphene	0.2 ÷ 2.2	Cançado at al. [51]
Oxygen plasma-etched graphene	≃0.1 ÷ 4	Childres et al. [52]
Fluorinated/anodic bonded graphene	≃2.3	Eckmann et al. [53]

### 3.3. Transmission Electron Microscopy

**Figure 4:**
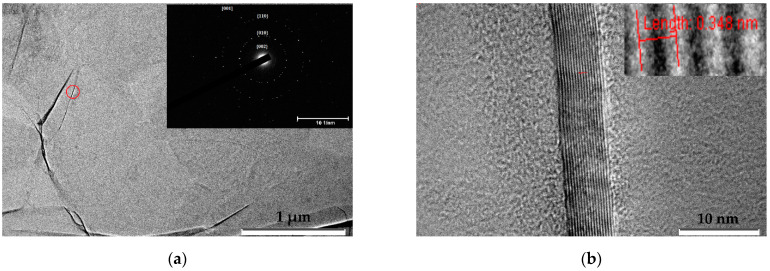
(**a**) TEM images of an initial unirradiated MLG. On the inset the diffraction pattern of a folded region was highlighted by the red circle; (**b**) High Resolution TEM images of the edge of a folded region. The inset shows enlarged image of a folded region indicating distance between graphene sheets of 0.348 nm.

**Figure 5:**
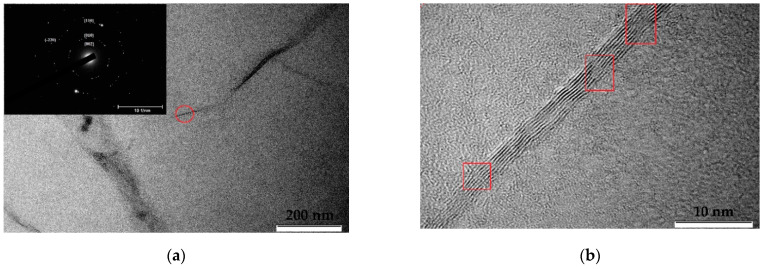
(**a**) TEM images of MLG irradiated by 30 keV (Ar_n_)^+^ at an ion beam dose of 10^14^ ion/cm^2^. The inset shows the diffraction pattern of a folded region highlighted by the red rectangles; (**b**) High Resolution TEM image of the edge of a folded region. Traces of penetration of argon clusters through MLG are highlighted by a red rectangle.

Both initial and irradiated MLG were deposited on ultrafine copper grids and were identified in a TEM (JEOL JEM 2100 operating at 200 kV). TEM results are seen in Figure 4 and Figure 5, respectively. Graphene layers have a wrinkled paper-like multilayer structure. TEM images revealed high-contrast areas indicating different graphene orientations; see Figure 4a and Figure 5a. The number of graphene layers in sheets was determined by inspecting the edges of a folded region; see Figure 4b and Figure 5b. The electron diffraction corresponding to these regions confirmed that it was polycrystalline graphene film; see Figure 4a and Figure 5a. Bright spots on the diffraction rings indicated large graphene crystallites. In the unirradiated sample Figure 4b, graphene layers had no disturbances or broken spots, and the distance between layers was rather uniform.

In Figure 5b that depicts irradiated MLG, the uniformity of layers is disturbed. However, upon closer examination of these areas, it can be seen that irradiation led to cross-linking of the layers in both the transverse and longitudinal directions; see Figure 5b. When graphene is irradiated, many defects appear in it, including structural defects leading to the formation of dangling bonds. We believe that covalent bonds between adjacent graphene layers can form due to these dangling bonds.

Our experimental result is consistent with the results of simulation. The joining of the overlapped graphene sheets by carbon ions beam irradiation was shown using classical molecular dynamic simulations in Ref. [54]. One of the important results obtained by the authors of this work is that the connection of two overlapping graphene sheets is attributed to ion irradiation, and not to the post-irradiation high-temperature annealing process. In the process of joining carbon nanotubes [55], it was also demonstrated that high temperature cannot provide the welding of nanotubes without irradiation. The idea of bonding graphene layers using irradiation with silicon ions using molecular dynamics simulation was proposed in Ref. [56]. The authors of this work noted that irradiation of the graphene structure, in addition to creating the bonds between individual planes, can also cause bonding between graphene stacks in the border regions.

**Figure 6:**
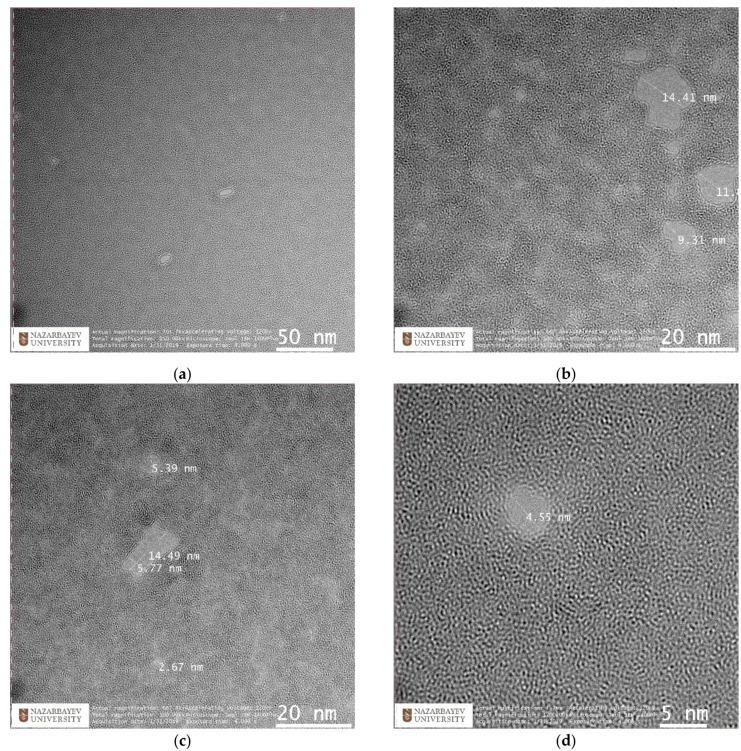
TEM and high resolution TEM (HRTEM) images of multiple nanopores (**a**–**c**) and a HR TEM image of a nanopore (**d**) on MLG irradiated with 30 keV cluster ions of (Ar_n_)^+^ (n ≈ 1000), at an ion dose of 10^14^ ion/cm^2^.

To directly observe the nanopores, a high-resolution transmission electron microscope (JEOL JEM-1400 Plus operated at 120 kV) was employed. The microscope is equipped with a digital image registration system based on Gatan OneView 16 MP camera. In Figure 6, TEM images of irradiated MLG with 30 keV Ar+ at ~10^14^ ion/cm^2^ are shown.

Some nanopores had a ring-like structure, and the nanopore edges were in fact quite smooth locally, although some pores, in TEM images, appeared to be irregularly shaped; see Figure 6c. The irregular shapes might be directly related to the shapes of the clusters that penetrated through the MLG during the bombardment. The characteristic number of atoms (molecules), n, in the Ar cluster varied between 2 and 10^4^. Clusters are formed in a typical expansion with a wide range of sizes. The average size of clusters can be adjusted by adjusting source–gas pressure, nozzle-throat diameter, electron ionization current, acceleration voltage, and the residual gas pressure in the ionizer. An Ar cluster ion beam with an ion current of about 100 μA was produced using a pressure of 4000 Torr for multilayer graphene irradiation in our experiment. Both beam intensity and average cluster size increased with source–gas pressure. At a source pressure of 4000 Torr, the average cluster size was more than 1000 atoms per cluster.

**Figure 7:**
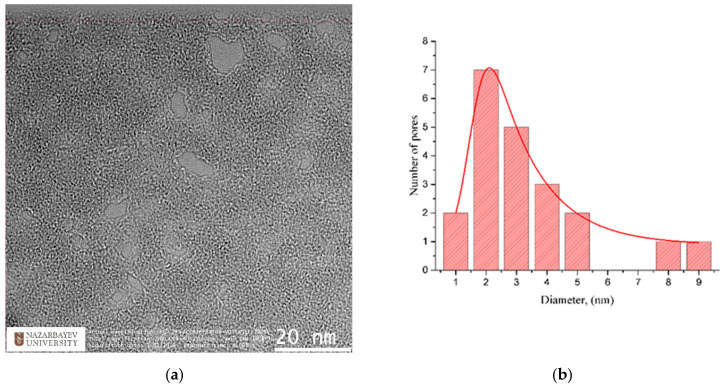
(**a**) TEM images of MLG irradiated by 30 keV (Ar_n_)^+^ at 10^14^ ion/cm^2^, containing 21 nanopores; (**b**) Histogram of the nanopore diameter distribution for typical irradiation conditions.

The pore size calculation was carried out using the ImageJ (https://imagej.nih.gov/ij/index.html, accessed on 21 December 2021) software package, which allows direct measurement of pores on TEM-images using a special ruler, and subsequently stores relevant data in a text file. Moreover, the pore-size distribution was captured using a standard graphical editor; see Figure 7. The predominant pore size in our sample was 2 nm, with an average diameter around 3 nm, as can be seen in the included histogram. Literature data on the sizes of nanopores in graphene structures vary greatly depending on their manufacturing method. For comparison, we cite several radiation methods, including gamma rays, focused beam and ultraviolet. In particular, nanoscale pores with average size of ∼3 nm were generated across 10 μm thick graphene oxide bucky-papers, by gamma ray irradiation in hydrogen [57]. Nanopores with a diameter of <10 nm were fabricated in graphene layers by Fox at al. [58], using a low-energy electron beam. Celebi et al. [59] used a focused ion beam to produce pores with diameters between less than 10 nm and 1 μm, in double-layer graphene. In Ref. [60], pores with a diameter of 0.40 ± 0.24 nm in graphene were fabricated by ion bombardment and oxidative etching. In Ref. [61] graphene nano-meshes with a pore size of ∼200 nm were obtained using UV-assisted photodegradation of the graphene oxide sheets.

## 4. Conclusions

Argon gas cluster ions were used for experimental fabrication of pore production on multilayer graphene. The structure of unirradiated and irradiated samples of MLG was studied using Raman spectroscopy and transmission electron microscopy. Raman spectroscopy of irradiated samples demonstrated a defects’ increase in MLG irradiated with 1 × 10^14^ ions/cm^2^. Transmission electron microscopy showed that irradiation of MLG with argon cluster ions leads to cross-linking between the individual planes and graphene stacks in the border regions, and that also nanopore appear in it. Some nanopores had a ring-like structure and, generally, nanopore edges were quite smooth locally, although some pores in the TEM images appeared to be irregularly shaped. Pore sizes varied from a few to a few tens of nm. Statistical analysis of some TEM images, acquired at different locations of the MLG, indicated an average pore diameter of 3 nm, with the predominant size corresponding to 2 nm. In summary, it can be argued that irradiation of MLG with GCIB is one of the promising directions for creating lightweight three-dimensional nanomaterials. The clear advantages of this approach include its speed and resulting cleanliness, whereas the lack of periodicity in pore location and their low density on the surface are issues that need to be tackled in future studies. It is expected that appropriate variation of irradiation conditions can control the pore location and/or density.

## Figures and Tables

**Table 1:** Parameters of Tersoff potential used in MD simulations.

Parameter	Value/Coordinates
Lattice constant	1.421 A
Lattice constant	(3, 0, 0)
Lattice constant	(0, 1.732, 0)
Lattice constant	(0, 0, 2.357)
Basis atom 1	(0, 0, 0)
Basis atom 2	(0.333, 0, 0)
Basis atom 3	(0.5, 0.5, 0)
Basis atom 4	(0.833, 0.5, 0)

**Table 2:** Parameters of Lennard–Jones potential for argon-argon interaction.

Interaction	ϵ (A)	σ (kcal/mol)	Cutoff (A)
Ar–Ar	0.238	3.4	7.65

**Table 3:** Parameters of Buckingham potential for argon–carbon interaction.

Interaction	A (kcal)	ρ (A)	C
C-Ar	74,569.79	0.2863	0.1 × 10^−9^

## Data Availability

The data that support the findings of this study are available on request from the corresponding author, Z.I.
